# The role of ADAMTS genes in preeclampsia

**DOI:** 10.4274/tjod.57701

**Published:** 2016-09-15

**Authors:** İrem Eda Gökdemir, Özlem Evliyaoğlu, Buğra Çoşkun

**Affiliations:** 1 Yenimahalle Training and Research Hospital, Clinic of Obstetrics and Gynecology, Ankara, Turkey; 2 Zekai Tahir Burak Women’s Health Training and Research Hospital, Clinic of Obstetrics and Gynecology, Ankara, Turkey; 3 Dr. Nafız Körez Sincan State Hospital, Clinic of Obstetrics and Gynecology, Ankara, Turkey

**Keywords:** ADAMTS genes, placentation, preeclampsia

## Abstract

Preeclampsia is a complex disease that increases both maternal and fetal morbidity and mortality in both developed and developing countries. It complicates around 5-10% of all pregnancies..The pathophysiology of preeclampsia includes both maternal and fetal/placental factors. Implantation of embryo and placentation are crucial steps for development of pregnancy involving trophoblast invasion. Abnormalities of spiral artery invasion, trophoblast function, inflammatory process, and biologic functions of angiogenic/anti-angiogenic factors early in pregnancy result in pregnancy diseases, including preeclampsia. *ADAMTS* genes are members of the family of matrix metalloproteinase, which have important tasks in extracellular matrix (ECM) degradation and repair processes. The roles of *ADAMTS* in preeclampsia may include regulation of spiral artery invasion and ECM arrangement of the placenta.

## INTRODUCTION

**Figure 1:**
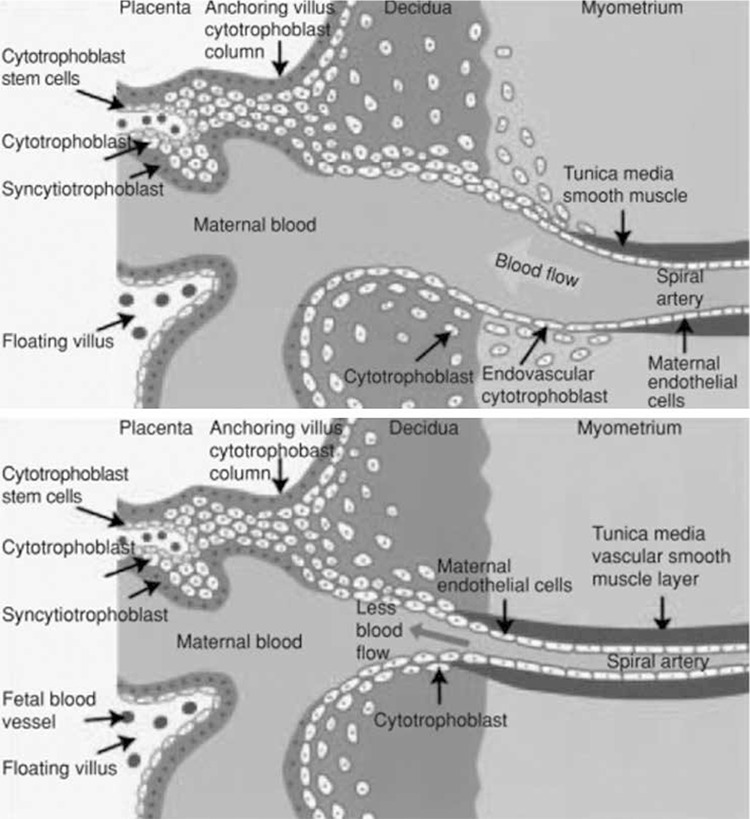
Normal placentation-pseudovasculogenesis (upper panel) and abnormal placentation in preeclampsia (lower panel)^(11)^

Preeclampsia is defined as the clinical condition associated with hypertension (systolic blood pressure ≥140 mm Hg or diastolic blood pressure ≥90 mm Hg) and proteinuria or end-organ dysfunction in a woman who was normotensive before 20 weeks gestation(1,2). Hypertensive diseases in pregnancy account for 16% of maternal deaths in developed countries^(1)^. According to data in the United States in 2010, 12% of pregnancy-associated maternal deaths are due to preeclampsia and eclampsia^(1)^. The main characteristics of hypertensive disorders and fetal growth restriction in pregnancy are gestation-specific restructuring of spiral arteries and defects in trophoblastic invasion^(3,4,5)^. In normal implantation, highly invasive trophoblast cells migrate to the decidua and myometrium and invade the endothelium of spiral arteries along with the muscularis tunica media. Smooth muscle structures at the distal part of uterine spiral arteries disappear. Terminal branches of the uterine artery transform into vessels that bear high capacity and low resistance, and provide the blood flow needed for development of the placenta^(6,7)^. Although gestation-specific restructuring of the spiral arteries begins at the end of the first trimester and is completed by the 18-20^th^ weeks, it is not known when trophoblastic invasion is terminated. Although they infiltrate the decidual spiral arteries, cytotrophoblasts cannot penetrate into the myometrium and pseudovasculogenesis does not occur^(8,9)^. This in turn leads to undesirable conditions such as placental hypoperfusion, placental infarction and atherosclerosis, fetal demise during the second trimester, placental abruption, preeclampsia, intrauterine growth restriction (IUGR), preterm labor, and premature rupture of membranes^(1,10,11)^. Invasion of spiral arteries by trophoblasts, release of special matrix metalloproteinases, and embodiment of the extracellular matrix (ECM) structure are necessary^(12)^. ECM has an active role in regulating cellular activity and behaviors such as shaping the cell, differentiation, division and programmed cell death. Matrix metalloproteinases are a member of the *ADAM* and *ADAMTS* zinc-dependent proteinases family. *ADAMTS* degrade molecules that act on regulation of the tissue microenvironment. Some of these molecules belong to the ECM (collagen, proteoglycan, and many other glycoproteins), and others do not (receptors, growth factors, and cytokines). It was shown that spiral artery invasion was limited in preeclampsia (Figure 1).

Changes in the ECM in the placentas and umbilical cords of the pregnant women with preeclampsia are different than those in normal pregnancies; however, the etiology is not yet clear^(13,14,15)^. The roles of cellular adhesion molecules, angiogenic proteins, and the inflammation system on microvascular dysfunction are undeniable in patients with preeclampsia^(16)^. Impairment of trophoblastic cell differentiation accounts for the inability of spiral arteries to invade into trophoblasts. Cytokines, adhesion molecules, ECM metalloproteinases, and class 1b major histocompatibility complex molecules released during trophoblastic invasion of endothelial cells and changes in HLA-G expression act on trophoblast differentiation^(17,18)^.

Preeclampsia can result in maternal complications such as eclampsia, edema, hypertensive encephalopathy, stroke, kidney and liver failure, liver rupture, retinal detachment, blindness, disseminated intravascular coagulation, and death^(19)^; and fetal outcomes such as IUGR, oligohydramnios, asphyxia, prematurity, preterm labor, and perinatal death. Studies on biological markers are needed in order to understand the etiology of this disease and predict preeclampsia.

**Figure 2:**
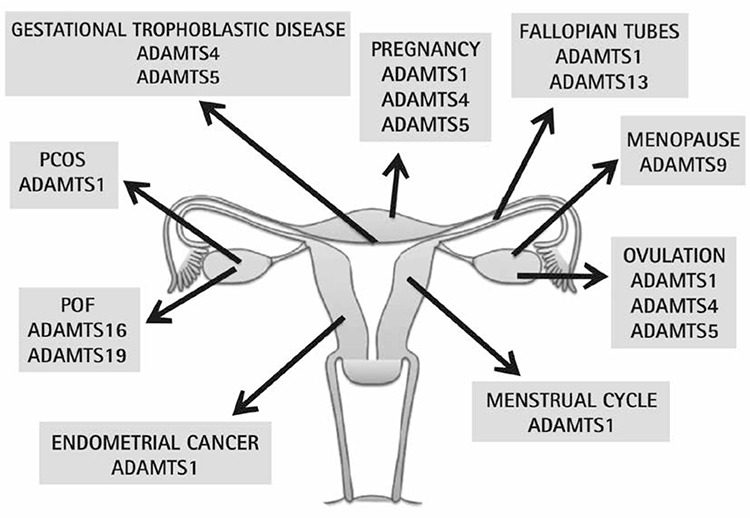
The role of the ADAMTS: A new biological marker candidates in physiological and pathological processes in female reproductive system^(22)^

*ADAMTS* genes were discovered in 1997 and were first defined by Kuno et al.^(20)^ as associated with colon cancer and inflammation. *ADAMTS* proteinases currently involve many physiologic and pathologic processes such as the those of the female reproductive system (Figure 2)^(21,22)^.

*ADAMTS* play a role in events such as restructuring of tissue, coagulation, angiogenesis, degradation of the ECM and basal membrane, and tumoral cell invasion and metastasis^(23,24)^. *ADAMTS* should be expressed, and the ECM must be degraded and formed so that trophoblasts can invade maternal tissues and spiral arteries. Invasion of the ECM is provided by the release of complicated proteases. *ADAMTS*-1, -2, -4, -6, -7, -9, and -12 subtypes are expressed during the first trimester in human placenta^(25)^. In addition, *ADAMTS*-1, -4, -5, -6, -7, -9, and -10 mRNA expressions were detected in term placenta^(26,27,28,29,30)^. Therefore, it is important to interpret the molecular organization and function of *ADAMTS*.

**Figure 3:**
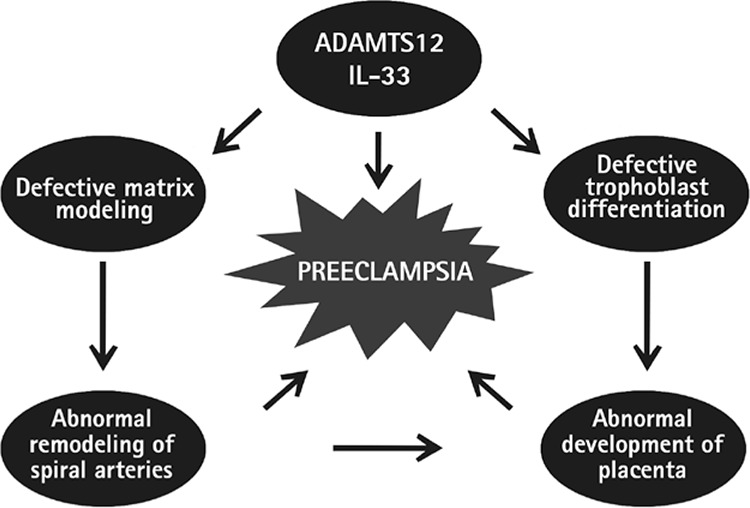
Deficiency of ADAMTS-12 may cause defective trophoblast differentiation and matrix reshaping, abnormal remodeling of spiral arteries and finally abnormal development of the placenta that induce preeclampsia^(34)^

*ADAM*-12 is among factors that predict preeclampsia^(1)^. One of two types of *ADAM*-12 is secreted (ADAM-12s), which interferes with the function of insulin-like growth factor- binding protein 3 (IGFBP-3) and IGFBP-5, which in turn leads to the development of preeclampsia^(31)^. The difference between *ADAMTS* and *ADAM* is the thrombospondin (TSP) portion, which resides at the molecular level. TSP is the ECM adhesion glycoprotein secreted from thrombocytes and is an angiogenesis inhibitor^(32)^. *ADAMTS*-12 is expressed in precedence by extravillous trophoblasts as compared with other ADAMTS^(33)^. Independent from the proteolytic activity of the enzyme, loss or decrease of *ADAMTS*-12 function diminishes the trophoblastic invasion. *ADAMTS*-12 regulates the cell invasion by regulating the avβ3 integrin heterodimer function and expression, and controls the trophoblast invasion by affecting the in vitro level of *ADAMTS*-12-transforming growth factor-β1 and interleukin-1β^(33)^. As a result, compared with other *ADAMTS*, *ADAMTS*-12 is secreted in precedence from the placental tissues and increases the invasion of trophoblasts. Deficiency of *ADAMTS* enzymes leads to several pregnancy complications, mainly preeclampsia. Eda Gokdemir et al.^(34)^ provided evidence that *ADAMTS*-12 levels were significantly decreased in the serum of patients with preeclampsia. Deficiency of *ADAMTS*-12 may cause defective trophoblast differentiation, abnormal remodeling of spiral arteries, and abnormal development of the placenta, which induces preeclampsia. Thus, *ADAMTS* proteinases play crucial roles in a variety of normal and pathophysiologic processes of placentation (Figure 3).

Daglar et al.^(35)^ studied placental levels of *ADAMTS*-12 to determine whether levels of enzymes differed among early-onset and late-onset severe preeclampsia. Early-onset preeclampsia was more likely associated with placental factors in impaired implantation and invasion than maternal factors. However, there were no significant differences in *ADAMTS*-12 levels between the groups. Also, *ADAMTS* genes are associated with other diseases such as ovarian cancer, polycystic ovarian syndrome, and premature ovarian failure^(36,37)^. These genes play multiple roles in male and female fertility^(38)^.

## RESULT

Preeclampsia is one of the important complications of pregnancy. Early prediction of the disease is crucial in order to prevent maternal and fetal morbidity and mortality. A simple, cost-effective test performed in pregnant women with high-risk of developing preeclampsia would have significant effects on maternal and fetal morbidity and mortality of this disease. In *ADAMTS* function deficiency, impairments in differentiation of trophoblasts, invasion of spiral arteries, angiogenesis, and ECM restructuring ensue. Implantation failure can lead to abortion, preterm labor, early membrane rupture, pregnancy-associated hypertensive diseases, and preeclampsia.

## CONCLUSION

*ADAMTS* genes are potential candidates in the pathophysiology of preeclampsia. Further studies are needed to determine whether these molecules can predict preeclampsia.
